# Thermal Characterization and Recycling of Polymers from Plastic Packaging Waste

**DOI:** 10.3390/polym17131786

**Published:** 2025-06-27

**Authors:** Maria-Anna Charitopoulou, Stavri Koutroumpi, Dimitrios S. Achilias

**Affiliations:** Laboratory of Polymer and Color Chemistry and Technology, Department of Chemistry, Aristotle University of Thessaloniki, 541 24 Thessaloniki, Greece; ccmariaa@chem.auth.gr (M.-A.C.); koutroumpestaure@gmail.com (S.K.)

**Keywords:** plastic packaging waste, identification, recycling, pyrolysis

## Abstract

Today, the global production of plastic packaging reaches a million tons annually, resulting in significant amounts of plastic waste in the environment, which causes serious pollution issues and negatively affects the health of all living beings. However, the recycling rate for plastic packaging waste in Europe currently remains limited (~38%). With this in mind, this study focuses on the collection, characterization, and recycling, through pyrolysis, of 23 random plastic samples collected from food and non-food packaging waste in Greece. The samples were analyzed using thermal characterization techniques, such as Differential Scanning Calorimetry (DSC) and Evolved Gas Analysis (EGA), in conjunction with FTIR spectroscopy to gather important information and identify the polymers present in each sample. Furthermore, the samples underwent pyrolysis, resulting in valuable products such as the monomers styrene or ethylene, along with other useful secondary compounds, including benzoic acid, depending on the polymer type of each sample. The most prevalent polymer identified was PE (35%), while the remaining samples consisted of PET (22%), PP (22%), and PS (17%); only one sample was a blend of PE/PP. DSC facilitated the identification of the polyethylene type (LDPE, HDPE, or LLDPE).

## 1. Introduction

The production of plastic materials, particularly plastic packaging, has skyrocketed globally over the past few decades. It is estimated that since the 1950s, more than 8 billion tons of plastic have been produced, a large percentage of which has been discarded into the environment or ended up in landfills [1]. The popularity of plastics stems from their properties—they are lightweight, durable, flexible, and inexpensive to produce, enabling their use in numerous applications, including packaging, the automotive industry, electric and electronic devices, etc. [2].

Plastic materials are widely used in packaging; specifically, plastic packaging accounts for almost 40% of the total plastic production globally and in Europe [3,4]. It is estimated that approximately 60% of all plastic packaging is used for food and drinks [3], while the remaining percentage is for non-food packaging. The most abundant polymers used in plastic packaging include low-density polyethylene (LDPE), linear low-density polyethylene (LLDPE), high-density polyethylene (HDPE), polypropylene (PP), poly(ethylene terephthalate) (PET), and polystyrene (PS) [5].

The increased consumption of plastic packaging and its short lifespan (most of it being single-use products) result in a growing amount of plastic packaging waste [6]. These properties make them extremely difficult to manage after their use. Their high durability also poses a significant drawback, as they are characterized by low biodegradability. This feature, along with improper handling, can lead to serious environmental issues, as they can accumulate in soils, oceans, and freshwater [2,7]. For example, a simple plastic bottle can take hundreds of years to fully decompose in the natural environment. In the meantime, it breaks down into microplastics, which enter the food chain and pose a threat to both ecosystems and human health [8]. Additionally, the incineration of plastic waste—a practice still widely used in several regions—produces toxic compounds that contribute to air pollution and climate change [9].

The accumulation of plastic waste in terrestrial and marine environments currently represents one of the most urgent environmental issues globally. Recycling is proposed as a key strategy for reducing pollution and promoting the circular use of materials. However, traditional mechanical recycling of plastics faces significant limitations. Many plastic products are multi-layered or contain additives and contaminants that make their recycling technically challenging or economically unviable. Moreover, the quality of the recycled product is often lower, which limits its use in high-end applications [10].

The significance of chemical recycling as a complementary and alternative technology now becomes apparent. In order to create new materials or fuels, chemical recycling attempts to break down polymers into monomers or other valuable chemical components [11]. Pyrolysis is a thermochemical process that breaks down polymers at high temperatures without oxygen, making it one of the most promising chemical recycling techniques. Useful substances, including hydrocarbons, oils, and gaseous fuels, can be extracted during pyrolysis and utilized as raw materials for a variety of industrial processes or as energy sources [12].

Compared to mechanical recycling, pyrolysis allows for the processing of a greater variety of plastic waste and the production of higher-value products. Additionally, it can aid in decreasing the dependence on petroleum and other non-renewable raw materials, thus supporting the circular economy [13].

Scientific and technological interest is increasingly focused on the investigation and implementation of pyrolysis as a method of chemical recycling, with emphasis on evaluating operational parameters, the quality of the resulting products, and its potential for industrial applications, to establish it as a valuable tool in addressing the plastic crisis.

For the identification of the polymer type in plastic packaging wastes, the thermal analytical technique of Differential Scanning Calorimetry (DSC) can be applied. This method enables the recording of characteristic thermal transitions, such as the melting temperature (T_m_) and the glass transition temperature (T_g_), which are distinctive for each type of polymer. The analysis of the calorimetric curves contributes to the identification of the polymeric material of each sample, as well as to the assessment of its thermal behavior. The use of DSC is important for both the qualitative and quantitative analysis of complex plastic materials [14].

**Figure 1:**
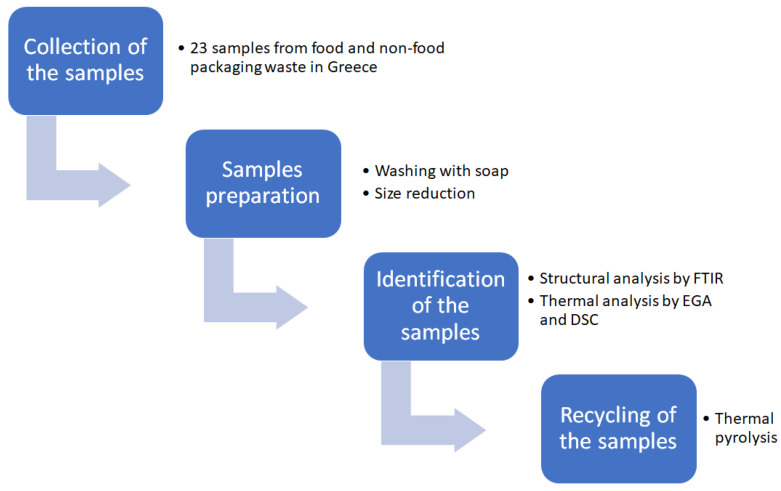
Flow diagram of the steps applied during this work.

The target of this work, on one hand, is to highlight the use of thermal analysis techniques to identify different types of polymers in plastic wastes and, on the other, to investigate the thermochemical recycling of plastic packaging waste, via pyrolysis, aiming to recover valuable products from waste. The main goal of this study is to offer new insights and contribute to the global target of increasing the recycling rate of plastic packaging, so as to ensure a more sustainable future. For this reason, a great variety of plastic packaging waste (23 samples) that were used for food and non-food packaging applications were collected and analyzed by various techniques (FTIR, EGA, and DSC) in order to identify the polymer type in each sample and to evaluate their recycling via pyrolysis. The plastic wastes used in this investigation were selected based on their abundance in everyday household plastic packaging. They included representative items, both food and non-food packaging, from different types of polymers. A schematic diagram that summarizes the experimental workflow is presented in Figure 1.

## 2. Materials and Methods

### 2.1. Materials

**Figure 2:**
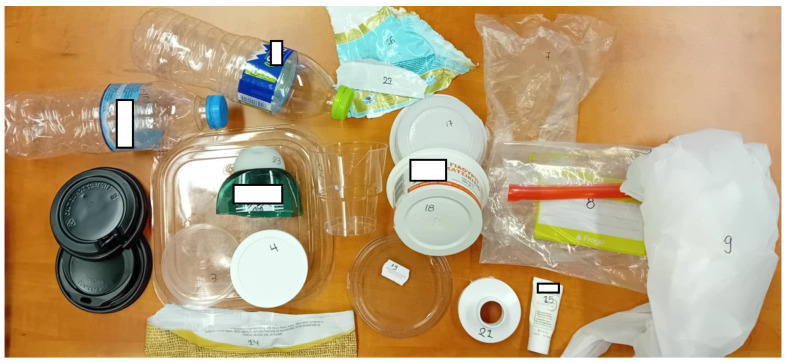
An overview of all samples studied.

During this study, 23 random plastic waste products (Figure 2) were collected from food (mainly) and non-food packaging household waste in Greece. It should be noted that since all plastic packaging waste samples investigated initially were thoroughly cleaned (washed with water and/or soap) to remove any food or other residues, they were wiped in order to dry them out. Afterwards, using hand-cutting tools, the samples were cut into small parts (size reduction) that were appropriate for the methods that would be applied.

Table 1 presents in detail all samples used, along with their names, categories, suspected polymer type, and a description of each sample.

### 2.2. Methods

After their preparation (cleaning, drying, and size reduction), samples were subjected to various techniques, such as FTIR analysis. Additional characterization of the samples also took place by applying thermal analysis techniques, such as DSC and EGA. Finally, the valorization–recycling of the samples was achieved via a thermochemical recycling method (i.e., pyrolysis), aiming to obtain either monomers or valuable secondary products. It should be underlined that, prior to the measurements, a calibration process took place. For this reason, standard, pure PET, PE, PS, and PP samples were subjected to the aforementioned techniques before measuring the real plastic waste samples, applying the same conditions (e.g., heating rate, etc.). The importance of this step was twofold: firstly, to calibrate the instruments in order to ensure better accuracy in the results obtained, and secondly, to be able to compare the new data (such as FTIR spectra) with the data obtained from the standard polymers, enabling their identification.

#### 2.2.1. FTIR Analysis

Fourier-transform infrared spectroscopy (FTIR) was applied in order to obtain the chemical structure of the unknown plastic samples by recording their IR spectra and, therefore, to distinguish the characteristic peaks of the functional groups of the polymers present in the samples, enabling the identification of the polymers.

FTIR analysis was conducted using an FTIR spectrometer, Spectrum One, from Perkin Elmer (Waltham, MA, USA), accompanied by analogous software. Spectra were obtained within the range of 4000–600 cm^−1^. The resolution of the equipment was 4 cm^−1^, while 16 scans per spectrum were applied for better accuracy.

#### 2.2.2. Evolved Gas Analysis (EGA)

Evolved Gas Analysis (EGA) was carried out, with the aim of gaining information about the decomposition temperature range of each sample and finding the optimum degradation temperature at which pyrolysis was finally held.

EGA was carried out on a pyrolyzer (EGA/PY-3030D Frontier Laboratories, Koriyama, Japan), and the purge gas was He. The sample mass during each measurement was ~0.5 mg. During EGA, a metal capillary tube was used, and the samples were heated within the range of 100–700 °C, applying a heating rate of 20 °C/min, under a satisfactory vacuum.

#### 2.2.3. DSC Analysis

Differential Scanning Calorimetry (DSC) was also applied in order to measure the glass transition temperature (T_g_), the crystallization temperature (T_c_), or the melting point (T_m_) of the samples, depending on the sample examined. The DSC results were very important in the case of the polyethylene samples, since they enabled the separation of LDPE, HDPE, and LLDPE.

DSC was performed using the Pyris 6 DSC instrument (PerkinElmer) under nitrogen flow. All samples (except those based on PET) were heated from 30 to 180 °C, at a rate of 20 °C/min. They were then cooled from 180 to 30 °C at the same rate of 20 °C/min, and finally, they were heated from 30 to 180 °C at a rate of 20 °C/min. For PET samples, the heating occurred from 30 °C to 280 °C at the same rate (i.e., 20 °C/min) and involved two heating cycles to estimate their melting temperature, as melting was observed above 200 °C (approximately 250 °C). All the thermal data used in the study were obtained from the second heating cycle.

#### 2.2.4. Recycling Method—Pyrolysis

Thermochemical recycling, and especially pyrolysis, was applied for the determination of the pyrolysis products, aiming to receive monomers (such as styrene, in the case of styrenic polymers) or other useful compounds, such as hydrocarbons, for instance, in the case of polyolefins (LDPE, HDPE, and PP) that can be used as fuels. It is worth mentioning that apart from obtaining valuable products, the composition of the pyrolysis products played a vital role in the final identification of the polymers present in the unknown plastic samples (along with the aforementioned methods applied).

Pyrolysis was held using an Ultra Alloy 5% diphenyl–95% dimethyl polysiloxane capillary, and the pyrolyzer (EGA/PY-3030D) (QP-2010 Ultra Plus, Shimadzu, Kyoto, Japan) was coupled with a gas chromatograph/mass spectrometer (Py-GC/MS) (QP-2010 Ultra Plus, Shimadzu, Kyoto, Japan). Flash pyrolysis (0.5 min) took place at the maximum degradation temperature that was received from EGA. The purge gas was He, and the sample mass was ~0.5 mg every time. The temperature program lasted 40 min. The chromatograms obtained after each measurement were subjected to interpretation through Shimadzu post-run software (NIST 17 Library, v.2.1). During the post-run analysis, only clear, intensive peaks were taken into account to ensure safe conclusions.

## 3. Results and Discussion

In this section, the most important results obtained are presented.

### 3.1. Spectroscopic Characterization

**Figure 3:**
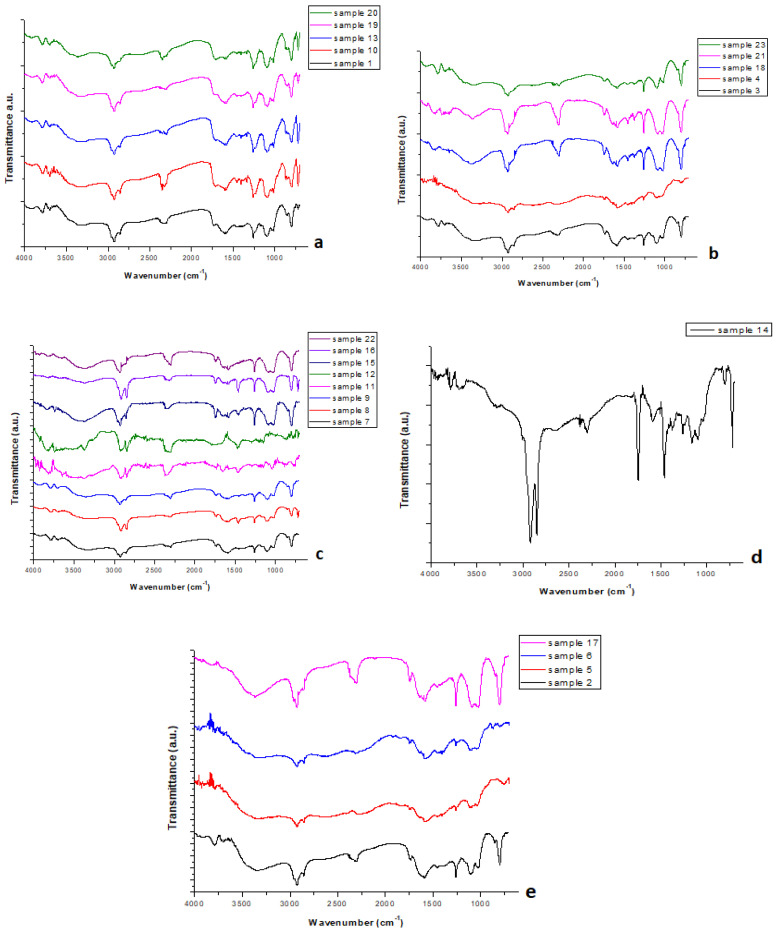
FTIR spectra for all samples studied. (**a**–**e**) include spectra from different samples, as reported.

Figure 3a–e present the FTIR curves obtained for all samples examined.

**Figure A1:**
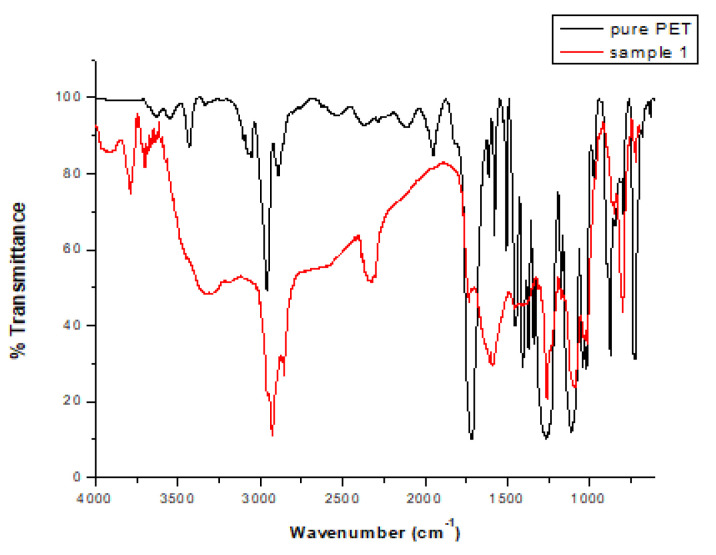
FTIR spectra of pure PET and sample 1 (PET).

Generally, the FTIR spectra of the samples presented in Figure 3a show a positive match for pure PET polymer (Figure A1). Therefore, these samples seem to be PET samples. This can be attributed to the wavenumbers observed at the main peaks. Specifically, the peaks at ~2924 and ~2856 cm^−1^ are due to the aromatic and aliphatic –C–H bond, the peak at ~1730 cm^−1^ is because of the ester carbonyl bond stretching (C=O), the peak at ~1580 cm^−1^ corresponds to aromatic stretching of the C=C bond, and the peaks at ~1252 cm^−1^ and ~1095 cm^−1^ are due to the ester group stretching (C-O) [15,16,17]. The aforementioned peaks are some of the main peaks obtained in the case of pure PET polymer.

The samples shown in Figure 3b,c seem to be polyolefin samples, e.g., PP or PE, since their structures are similar, taking into account that both of them comprise C and H only. Some of the main peaks observed in Figure 3b are the peaks at ~2931 and 2849 cm^−1^, along with those at ~1450 and 1374 cm^−1^, which are due to the C-H bond (of CH_3_ and CH_2_) and the peak at ~800 cm^−1^, which is owed to the C-C bond [18,19]. With regard to Figure 3c, some of the main peaks observed are at ~2921 and ~2848 cm^−1^, which are due to the C-H bond (of CH_3_ and CH_2_), the peak at ~1463 cm^−1^ is, again, owed to the C-H bond, and the peak at ~720 cm^−1^ is because of the rocking vibration of CH_2_ [20,21].

**Figure A2:**
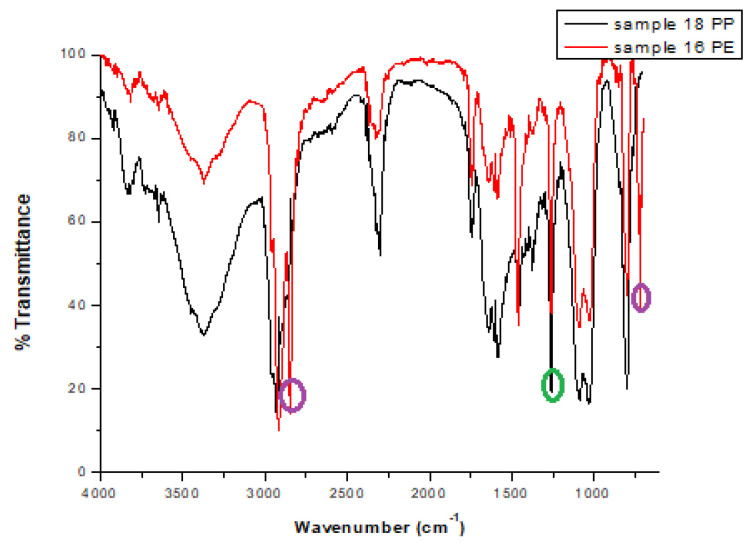
FTIR spectra of sample 18 (PP) and sample 16 (PE) for comparison.

**Figure A3:**
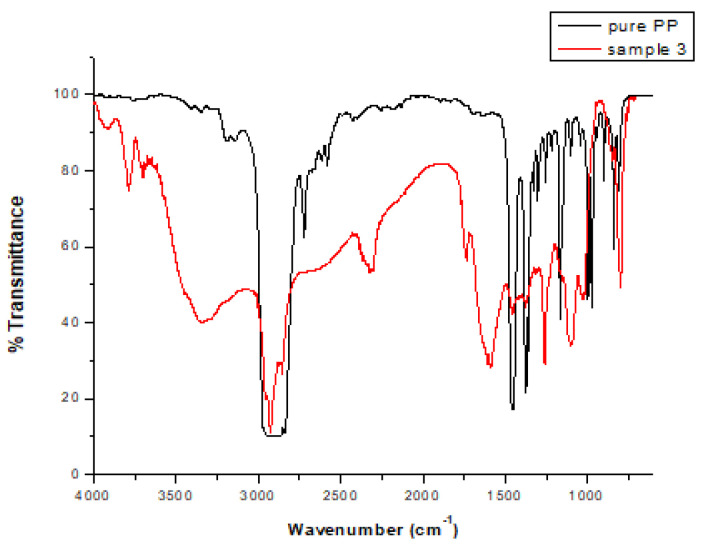
FTIR spectra of pure PP and sample 3 (PP).

**Figure A4:**
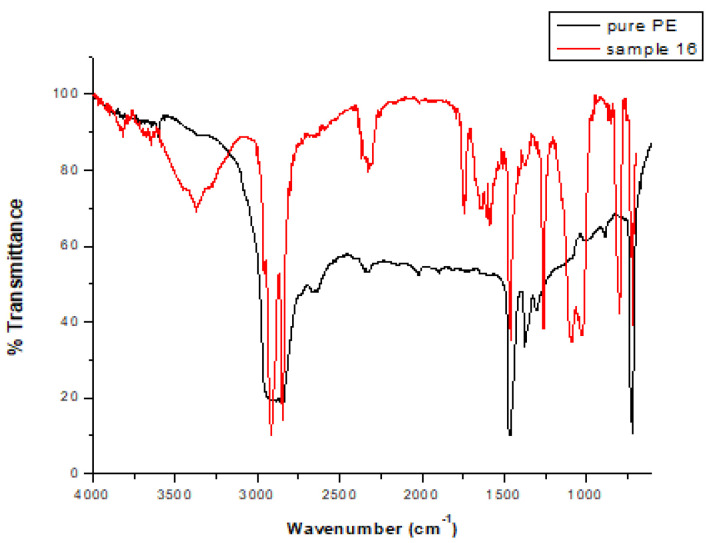
FTIR spectra of pure PE and sample 16 (PE).

Despite the similarities, it is assumed that the samples in Figure 3b consist of PP, while those in Figure 3c consist of PE due to some structural differences (Figure A2). Firstly, in the case of PE samples, the peaks within the range of 2800–3000 cm^−1^ are higher and sharper than those of PP samples. Specifically, the peak at ~2849 cm^−1^ is almost negligible in the case of the PP samples, whereas in the case of the PE samples, the peak at ~2848 cm^−1^ is of great intensity. Furthermore, the peak at ~1374 cm^−1^ is only found in the case of PP samples, while in the PE samples, there is no such peak. Additionally, the peak at ~720 cm^−1^, which is conspicuous in the case of the PE samples, is not present in the case of the PP samples. For a better understanding, Figure A3 presents the FTIR of pure PP along with the FTIR of sample 3, which was found to be the PP sample. Additionally, the FTIR of pure PE along with the FTIR of sample 16 is also presented (Figure A4) in order to show their positive match.

In Figure 3d, the main peaks observed are firstly those at ~2917 and 2850 cm^−1^, which can be attributed to the C-H bond and are found in many polymers (such as PET, PE, etc.). Another peak that was noticed is that at ~1467 cm^−1^, corresponding to the C-H bond again. The latter peak is found in polyolefin polymers (PE and PP). Additionally, a small peak at ~1374 cm^−1^ was present in the spectra, which can be found in PP polymers, because of the C-H bond. The last peak, but of great intensity, is that found at ~720 cm^−1^, which is due to the rocking vibration of CH_2_ and is usually observed in PE samples. Therefore, sample 14 appears to be a blend of more than one polymer, possibly PE and PP. Nevertheless, in this case, the application of additional methods seems to be mandatory in order to identify this unknown sample with accuracy.

**Figure A5:**
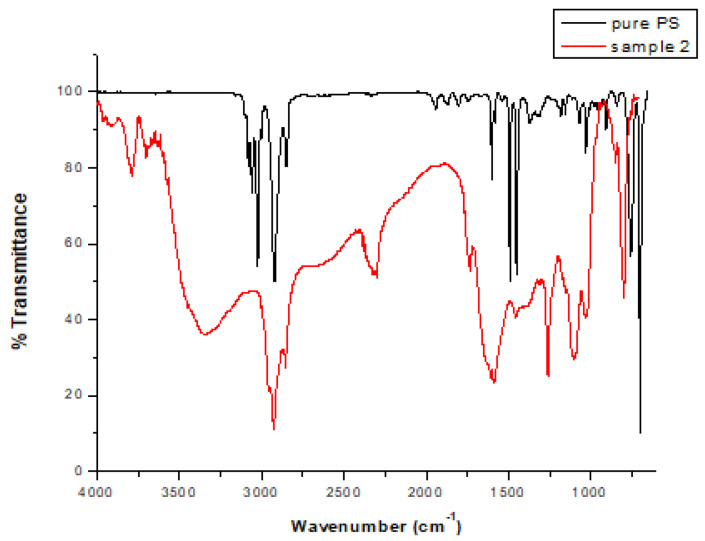
FTIR spectra of pure PS and sample 2.

The FTIR spectra of the samples presented in Figure 3e show a positive match to the pure PS polymer (Figure A5). Therefore, the curves presented in Figure 3e could be attributed to PS samples, due to the peaks obtained, including, for instance, peaks at ~2921 and 2848 cm^−1^ because of the aromatic and aliphatic C-H stretching, peaks at ~1602 cm^−1^ corresponding to the aromatic C=C stretching vibration, and peaks at ~760 cm^−1^ due to the ring deformation vibration [22,23].

From the spectroscopic characterization, it was clear that while important information can be gathered about the functional groups in various polymers, identifying the type of polyethylene is impossible. It should be noted that this information is crucial for further processing of these samples since the different types of polyethylene exhibit varying properties. Therefore, thermal characterization techniques were further employed.

### 3.2. Thermal Characterization via Evolved Gas Analysis

**Figure 4:**
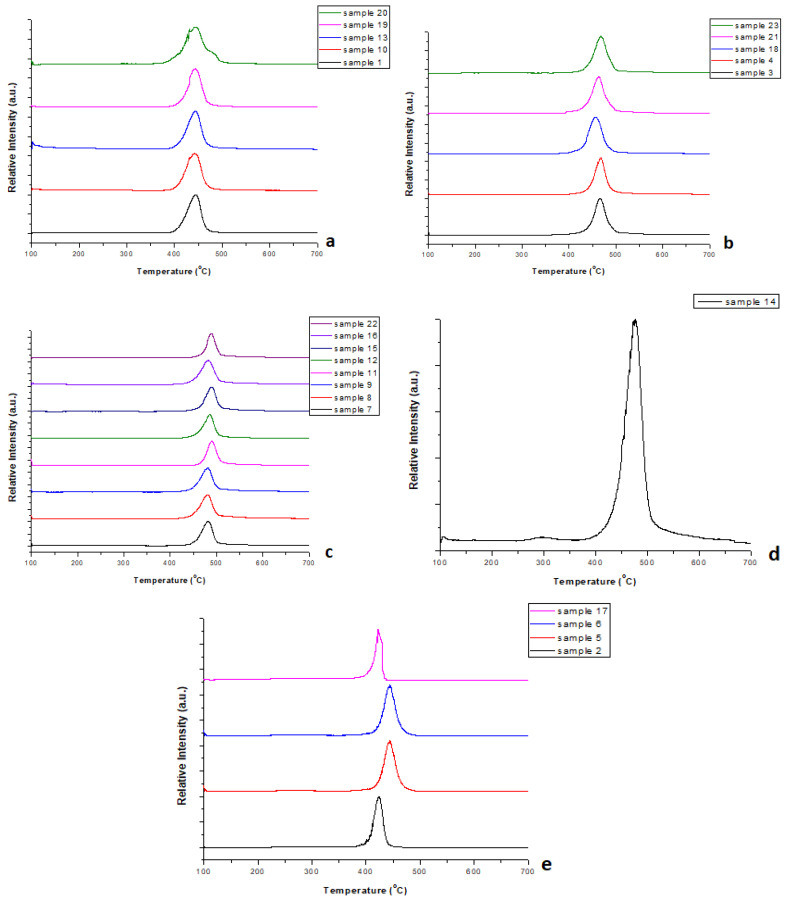
EGA curves obtained for all samples studied. (**a**–**e**) include data from different samples, as reported.

In Figure 4a–e, the EGA curves obtained for all samples are presented.

As observed in Figure 4a, the thermal degradation of the samples started at temperatures greater than 350 °C, reached the maximum degradation at about 440 °C, and was completed at ~500 °C. These temperature data are observed in PET polymers, especially the temperature of maximum decomposition (T_max_), according to [24].

According to Figure 4c, the thermal degradation of the samples was initiated at ~410 °C, reached the maximum degradation within the range of 480–491 °C, and extended up to ~525 °C. These temperatures are in accordance with those mentioned in the literature for PE polymers [25].

Similar observations, with small temperature differences, depending on the polymer type, were made for the rest of the samples examined (Figure 4b,e). For each sample, the temperature at which the maximum degradation occurred (T_max_) was selected as the pyrolysis temperature, with the aim of obtaining more valuable products. The T_max_ of each sample is presented in Table 2. It should be highlighted that the temperatures presented in Table 2 are the mean values, since EGA was conducted three times for each sample for better accuracy of the results.

### 3.3. Thermal Characterization via DSC

DSC results, along with those from the other methods employed, led to the final identification of the polymers present in the unknown samples. It should be emphasized that the application of DSC, in the case of the PE polymers, allowed for the precise identification of the sample types: high-density polyethylene (HDPE), low-density polyethylene (LDPE), and linear low-density polyethylene (LLDPE).

**Figure 5:**
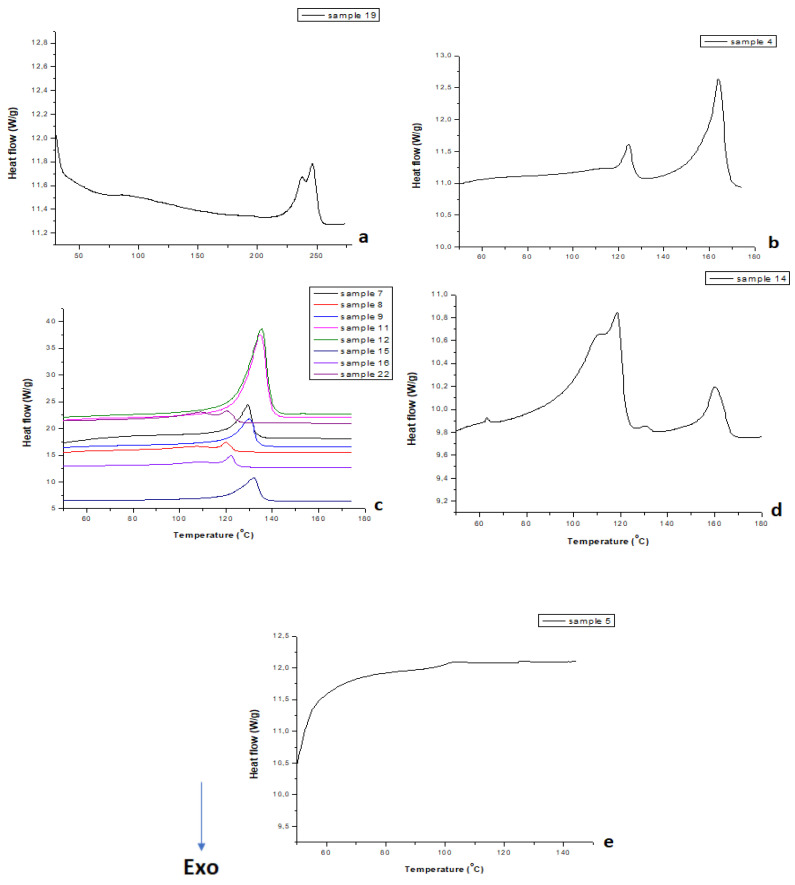
DSC curves of (**a**) a PET-based sample (sample 19), (**b**) a PP sample (sample 4), (**c**) all polyethylene samples, (**d**) a blend of PE/PP (sample 14), and (**e**) a PS sample (sample 5).

For samples 1, 10, 13, 19, and 20, it was observed that during the second heating cycle, their T_m_ was approximately 246–248 °C and their T_g_ was around 84–85 °C. These temperatures, according to data in the literature, are indicative of PET polymer [26]. Figure 5a presents an indicative DSC curve for one of the PET samples—sample 19.

In the case of samples 3, 4, 18, 21, and 23, it was noted that during the second heating cycle, their T_m_ was approximately 164–165 °C, which indicates PP polymer [27]. Again, an indicative DSC curve of one PP sample (sample 4) is shown in Figure 5b.

Regarding the PE samples—samples 7, 8, 9, 11, 12, 15, 16, and 22—DSC values (Figure 5c) demonstrated that some of them were HDPE, while others were LLDPE/LDPE. Table 3 presents the melting temperatures, the enthalpy of melting (ΔH_m_), and the crystallization temperatures for all PE samples, along with their precise identification.

Concerning sample 14, DSC values confirmed the co-existence of two polymers in a blend (Figure 5d). Specifically, during the second heating cycle, it was found that T_m1_ was approximately 118 °C and T_m2_ was around 160 °C, corresponding to LDPE and PP, respectively [28].

In the case of samples 2, 5, 6, and 17, it was found that during the second heating cycle, their T_g_ was around 99 °C, proving that they consist of PS (Figure 5e) [28].

### 3.4. Identification of the Samples Examined

The combination of all the analytical results (FTIR, DSC, and EGA) enabled the final polymer identification. Specifically, as already mentioned, via FTIR results, a first identification of the possible polymer present in each sample was achieved by comparing the FTIR spectra of the plastic waste samples with those of the pure polymers. In order to strengthen this first identification, or in the case of the PE samples, in order to identify the exact polyethylene type (which could not occur via FTIR), thermal characterization techniques were also employed. EGA results, along with DSC results, complemented the initial identification. Degradation temperatures, estimated by EGA, are characteristic of the polymers studied; for instance, the degradation temperature of PP is different from that of PS. This information supported the FTIR results. Last but not least, DSC provided important information, such as the melting and crystallization temperatures of the samples, enabling the further and final identification of all samples, along with the exact identification of the PE type (HDPE, LDPE, or LLDPE).

Therefore, based on the results obtained from all methods applied, the final identification of the polymer(s) present in each sample was achieved (Table 4). As can be observed, the majority of the samples were PE samples, with either one PE type (HDPE) or two PE types (LDPE/LLDPE). The rest of the samples consisted of PET, PP, PS, and a blend of PP/LLDPE. The presence of the aforementioned polymers is quite expected, based on the fact that they are considered the most abundant polymers used in plastic packaging [5].

### 3.5. Pyrolysis Results

Pyrolysis resulted in the production of useful compounds, including monomers and/or other secondary products. The composition of pyrolysis products varied depending on the polymer type of the sample examined. In Table 5, the most important products obtained for one sample of each category are presented.

**Figure 6:**
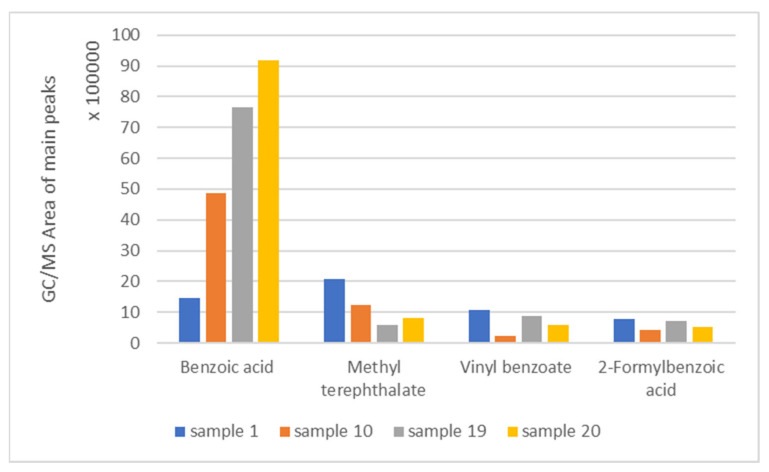
The total area of the most abundant pyrolysis products formed in the case of the PET samples.

According to the literature, the pyrolysis of PET results in the formation of oligomers, which can be used for making new copolymers and polyurethanes [29], as well as various esters of terephthalic and benzoic acid [24]. Therefore, the pyrolysis products of PET samples examined in this work, such as sample 1, which is mentioned in Table 5, are in accordance with those mentioned in the literature and can be considered as useful compounds. It should be highlighted that the products’ distribution does not change among the different PET samples. Specifically, the composition of the rest of the PET samples examined was the same as that of sample 1, as presented in Table 5, with differences in the peak areas. The aforementioned differences in the peak areas are shown in Figure 6, which shows that the peak areas vary from sample to sample. However, as noticed, the most abundant product in all cases examined is benzoic acid.

**Figure A6:**
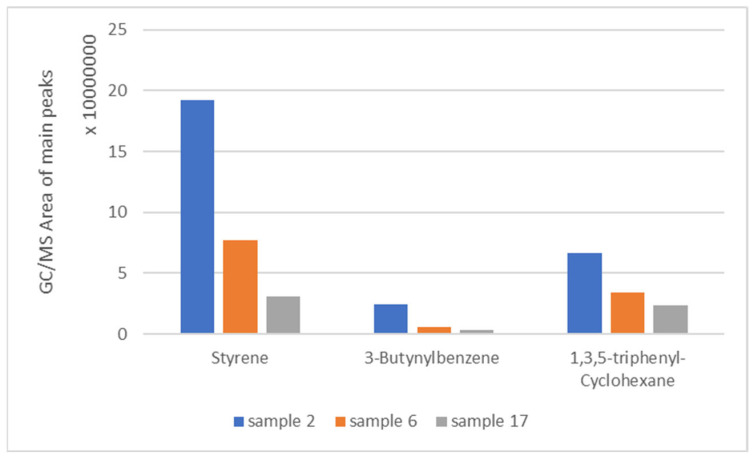
The total area of the most abundant pyrolysis products formed in the case of PS samples.

In the case of the PS samples examined during this study, such as sample 2, which is presented in Table 5, the monomer (styrene) is the main pyrolysis product, along with a styrene trimer (cyclohexane, 1,3,5-triphenyl-). These products are valuable, and their formation is in agreement with [30]. The distribution of the products is the same among the different PS samples, with some differences observed in the peak areas (Figure A6).

**Figure A7:**
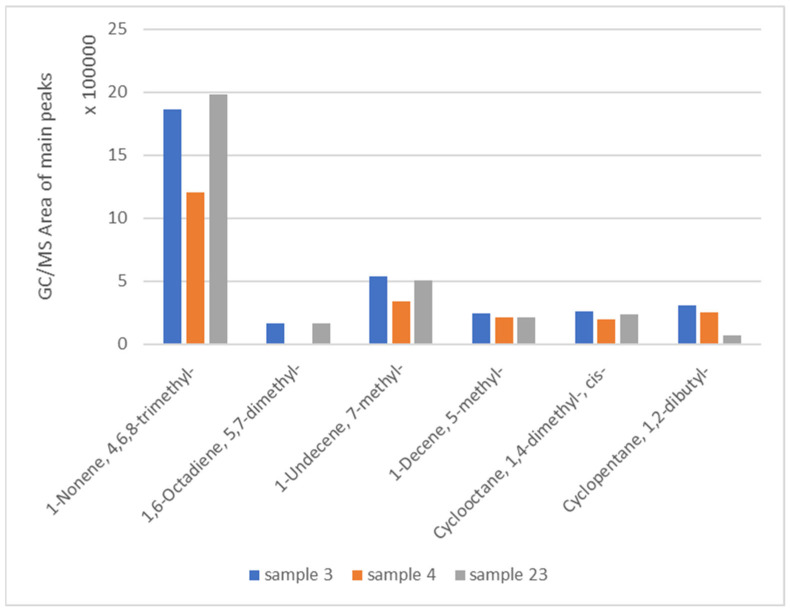
The total area of the most abundant pyrolysis products formed in the case of PP samples.

The thermal pyrolysis of PP samples (such as sample 3, presented in Table 5) leads to the formation of unsaturated aliphatic hydrocarbons, including alkenes (such as 7-methyl-1-undecene, 1-decene, 5-methyl, etc.) and alkadienes (such as 1,6-octadiene and 5,7-dimethyl-). Additionally, saturated hydrocarbons are formed, including cycloalkanes, such as cyclobutane, cyclooctane, 1,4-dimethyl-, cis- and 1,2-dibutylcyclopentane, etc. All these results are in agreement with the data in the literature, where it is mentioned that the thermal degradation of PP results in the formation of hydrocarbons [31,32]. The distribution of the products is again the same among the different PP samples, with differences observed in the peak areas (Figure A7).

**Figure A8:**
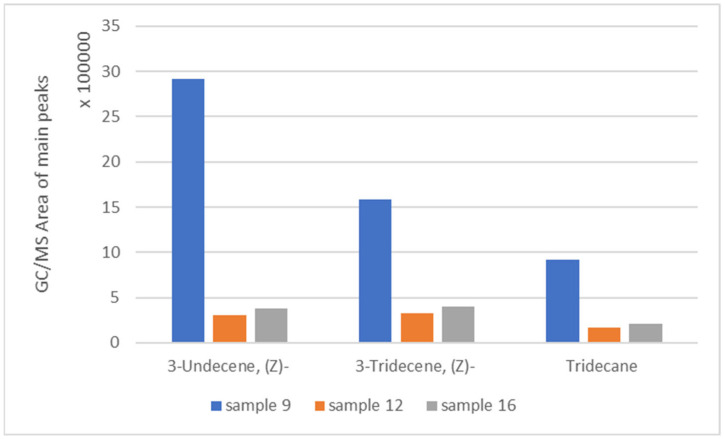
The total area of the most abundant pyrolysis products formed in the case of PΕ samples.

It is mentioned in the literature that the pyrolysis of PE leads to the formation of olefins and aliphatic and cyclic paraffins, while olefins can be within the range of 8 to 35 carbon atoms (C_8_ to C_35_) [25]. The thermal pyrolysis products of PE samples studied here, such as sample 9 shown in Table 5, were in agreement with the aforementioned products. Specifically, some of the most representative products obtained include octane, tridecane (paraffins/alkanes), 2-octene, 3-undecene, 3-tridecene (alkenes/olefins), 1,6-octadiene (dienes), etc. The vast majority of the compounds formed were alkenes composed of compounds from C_8_ to C_13_, which, according to the literature, are called alpha-olefin compounds and are in high demand in petrochemical industries, since they can be used as chemical feedstock for plastic and detergent manufacturing [33]. With regard to the distribution of the products, it is, again, the same among the different PE samples, with differences in the peak areas (Figure A8).

In the case of sample 14, which was found to be a blend of PP/LLDPE, it was observed that, again, the majority of the products formed were alkenes comprising eight to thirteen carbon atoms (C_8_ to C_13_), which, as already mentioned, are valuable in industries such as chemical feedstock for the production of new plastic products. Some of the most abundant products formed in the case of sample 14 are shown in Table 5.

## 4. Conclusions

During this study, 23 plastic waste samples from food and non-food packaging applications were analyzed. The results of this work are valuable because they demonstrate that, using FTIR, EGA, and DSC, a quick and easy, yet precise, characterization and exact identification of unknown plastics can occur. This study particularly highlights the importance of the DSC method, as it can be widely applied to plastic packaging waste where PE polymers are commonly found, enabling the identification of their exact PE type, which is crucial before handling—specifically recycling.

The findings from these techniques corroborate the existing literature, which states that PE, PET, PP, and PS are prevalent in plastic packaging applications. According to our findings, the most abundant polymer was PE, as 8 out of 23 samples were PE polymers, while 1 out of 23 was a blend of PP/PE. However, this trend cannot be generalized since a larger number of samples may be needed to ensure greater accuracy. It cannot be assumed with confidence that only these types of polymers in these percentages are present in various plastic packaging wastes in Greece. Therefore, future studies could examine more diverse plastic samples from packaging waste to confirm or refute this trend.

The thermal pyrolysis of the plastic waste samples was also explored. It was proven that pyrolysis led to useful products, either monomers that can be used for the production of the polymer, such as styrene in the case of PS plastics, or other secondary products, such as alkenes with eight to thirteen carbon atoms, in the case of PE plastics, which are valuable in industries such as chemical feedstock for the production of new plastic products. These findings are of paramount importance, since they show that the pyrolysis of plastic packaging waste may be a solution to the global environmental problem associated with increased amounts of this type of waste. Specifically, recycling via pyrolysis contributes to the circular economy model, where the waste is considered as a feedstock for a new process, such as the pyrolysis process. This is very important, taking into account that the take–make–waste approach (linear model), in which resources, after being extracted and transformed into products, are finally discarded as waste, is now replaced by sustainable approaches and such circular economy models.

Thus, future studies could investigate pyrolysis using bigger amounts of waste, in a larger-scale pyrolyzer, aiming to explore the efficiency of the method and the distribution of products. This can be considered a promising and challenging venture. Specifically, if pyrolysis could be applied as an industrial recycling method, then large amounts of monomers or other secondary compounds could be received, with the aim of producing new polymers, in a more sustainable way, without wasting natural resources, and in the meantime, the large volumes of plastic waste generated could be reduced. Nevertheless, this is no mean feat, since further investigation is required in order to overcome any difficulties associated with this venture, such as how to isolate the monomers or other valuable compounds from other compounds present in the pyrolysis fraction, or to examine the economic feasibility of this process.

## Figures and Tables

**Table 1:** Samples collected and their names.

Sample Description	Suspected Polymer Type	Sample Category	Sample Name
See-through cap (of food container)	PET	Food packaging	Sample 1
Disposable plastic cup (drink container)	PS	Food packaging	Sample 2
See-through cap	PP	Food packaging	Sample 3
White cap	PP	Food packaging	Sample 4
Black matte (coffee drink) cap	PS	Food packaging	Sample 5
Black glossy (coffee drink) cap	PS	Food packaging	Sample 6
Small transparent food bag	PE	Food packaging	Sample 7
Small transparent zip lock bag	PE	Food packaging	Sample 8
Transparent food bag	PE	Food packaging	Sample 9
Disposable water bottle (firm 1)	PET	Food packaging	Sample 10
Water bottle cap (firm 1)	PE	Food packaging	Sample 11
Water bottle cap (firm 2)	PE	Food packaging	Sample 12
Disposable water bottle (firm 2)	PET	Food packaging	Sample 13
Frozen food packaging	PE	Food packaging	Sample 14
Face cream packaging	PE	Non-food packaging	Sample 15
Diaper packaging	PE	Non-food packaging	Sample 16
Yogurt pot (firm 1)	PS	Food packaging	Sample 17
Yogurt pot (firm 2)	PP	Food packaging	Sample 18
Yogurt lid	PET	Food packaging	Sample 19
Shampoo bottle	PET	Non-food packaging	Sample 20
Face wash bottle	PP	Non-food packaging	Sample 21
Sanitary pad packaging	PE	Non-food packaging	Sample 22
Shampoo bottle cap	PP	Non-food packaging	Sample 23

**Table 2:** Maximum degradation temperature (T_max_) of each sample, based on EGA results. Mean values and standard deviation after 3 repetitions of the measurements.

Sample Name	T_max_ (°C)
Sample 1	444 ± 1
Sample 2	423 ± 2
Sample 3	466 ± 2
Sample 4	468 ± 3
Sample 5	440 ± 1
Sample 6	444 ± 1
Sample 7	482 ± 2
Sample 8	480 ± 1
Sample 9	481 ± 1
Sample 10	443 ± 1
Sample 11	489 ± 2
Sample 12	485 ± 3
Sample 13	444 ± 1
Sample 14	476 ± 2
Sample 15	491 ± 1
Sample 16	482 ± 1
Sample 17	433 ± 3
Sample 18	456 ± 3
Sample 19	444 ± 1
Sample 20	443 ± 1
Sample 21	463 ± 2
Sample 22	488 ± 2
Sample 23	468 ± 1

**Table 3:** Melting (T_m_) and crystallization (T_c_) temperatures and enthalpy of fusion (ΔH_m_) of all the PE samples and identification of their exact PE type, via DSC.

Sample Name	T_m_ (°C)	ΔH_m_ (J/g)	T_c_ (°C)	PE Type
Sample 7	129.5	141.6	115	HDPE
Sample 8	109; 120.3	114.4	107	LDPE/LLDPE
Sample 9	129.4	139.8	115	HDPE
Sample 11	134	160.2	117	HDPE
Sample 12	135	162.1	116	HDPE
Sample 15	132.2	144.2	115	HDPE
Sample 16	109; 122	122.5	108	LDPE/LLDPE
Sample 22	110; 121	118.7	105	LDPE/LLDPE

**Table 4:** Final identification of the samples.

Sample Name	Polymer Type
Samples 1, 10, 13, 19, and 20	PET
Samples 3, 4, 18, 21, and 23	PP
Samples 8, 16, and 22	LDPE/LLDPE
Samples 7, 9, 11, 12, and 15	HDPE
Samples 2, 5, 6, and 17	PS
Sample 14	PP/LLDPE

**Table 5:** The most representative products obtained for each category of the samples, after thermal pyrolysis.

Sample—Polymer Type	Pyrolysis Products	Pyrolysis Temperature—T_max_ (°C)
Sample 1—PET	Benzoic acid (8.7 min); Methyl terephthalate (13.5 min); Vinyl benzoate (17.8 min); 2-Formylbenzoic acid (21.6 min)	444
Sample 2—PS	Styrene (2.9 min); 3-Butynylbenzene (13.4 min); Cyclohexane, 1,3,5-triphenyl- (20.2 min)	423
Sample 3—PP	Cyclobutane (1.3 min); 2-Octene (2.3 min); 1-Nonene, 4,6,8-trimethyl- (8.2 min); 1,6-Octadiene, 5,7-dimethyl- (9.1 min); 1-Undecene, 7-methyl- (10.9 min); 1-Decene, 5-methyl- (11.8 min); Cyclooctane, 1,4-dimethyl-, cis- (14.1 min); Cyclopentane, 1,2-dibutyl- (20 min)	466
Sample 9—PE	1,6-Octadiene (6.5 min); Octane (6.8 min); 2-Octene (8.1 min); 3-Undecene, (Z)- (9.4 min); 3-Tridecene, (Z)- (10.7 min); Tridecane (14.2 min)	481
Sample 14—PE/PP Blend	2-Octene (2.2 min); 1-Nonene (8.2 min); 3-Decene (9.3 min); 4-Dodecene, (E)- (10.6 min); Undecane, 4-methyl- (10.7 min); 3-Tridecene, (Z)- (10.9 min); 3-Tetradecene, (E)- (16.0 min); Tridecane, 3-ethyl- (16.1 min)	476

**Figure A9:**
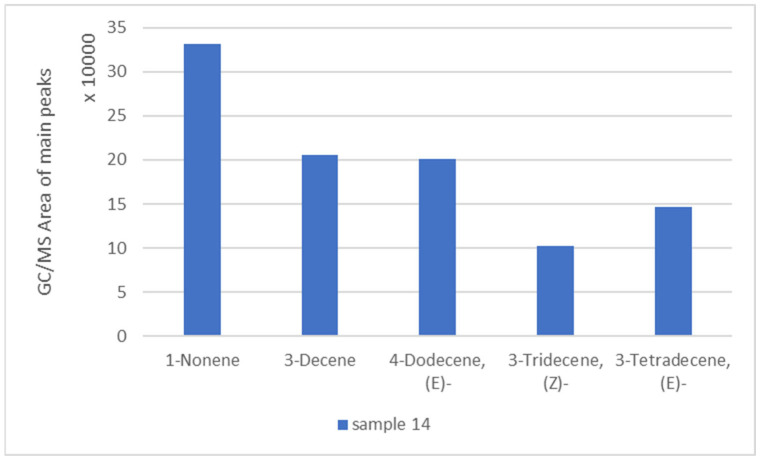
The total area of the most abundant pyrolysis products formed in the case of the PP/LLDPE sample.

## Data Availability

The original contributions presented in this study are included in the article. Further inquiries can be directed to the corresponding authors.

## Appendix A

In Figure A1, the FTIR spectra of pure PET and sample 1 are presented. As can be observed, the sample’s spectrum shows a positive match to the pure PET polymer spectrum. The same observation was made for samples 10, 13, 19, and 20.

In Figure A2, the FTIR spectra of a PE and PP sample are presented for a better explanation of the differences in their FTIR spectra, which enabled the identification of the samples. The circles in purple show the differences due to PE, while the green circle shows the differences due to PP.

In Figure A3, the FTIR spectra of pure PP and sample 3, which consists of PP, are presented. As can be observed, the sample’s spectrum shows a positive match to the pure polymer spectrum, and the same observation was made for samples 4, 18, 21, and 23.

Figure A4 presents the FTIR spectra of pure PE and sample 16, which is found to be a PE sample. As can be observed, the spectrum of sample 16 shows a positive match to the pure PE spectrum. The latter observation was also made for samples 7, 8, 9, 11, 12, 15, and 22.

Figure A5 shows the FTIR spectra of pure PS and sample 2, which is a PS sample. The spectrum of sample 2 presents a positive match to the pure PS spectrum. This observation was made for samples 5, 6, and 17 too.

Figure A6 presents the peak areas of the pyrolysis products of different PS samples, proving that the total area of each compound formed varies from sample to sample, while the monomer, styrene, is the most abundant product in all PS cases examined.

Figure A7 presents the peak areas of the pyrolysis products of different PP samples, showing again that the total area of each compound formed varies among the different samples. With regard to sample 4, one difference is the fact that the peak of 1,6-Octadiene, 5,7-dimethyl- is not presented in Figure A7, since this peak was almost negligible.

In Figure A8, the peak areas of the pyrolysis products of different PE samples are presented. It is obvious that the total area of each compound formed varies among the different samples.

In Figure A9, the peak areas of the pyrolysis products of the PP/LLDPE blend are shown.
